# Methodological study on simultaneous detection of 6 tumor invasion and metastasis markers including MMP-9 by microfluidic chip-based magnetic particle immunofluorescence assay

**DOI:** 10.1371/journal.pone.0351313

**Published:** 2026-06-23

**Authors:** Junhao Li, Yiran Wang

**Affiliations:** 1 School of Public Health, Xinjiang Second Medical College, Karamay, Xinjiang Uygur Autonomous Region, China; 2 School of Clinical Medicine, Xinjiang Second Medical College, Karamay, Xinjiang Uygur Autonomous Region, China; The Ohio State University, UNITED STATES OF AMERICA

## Abstract

**Objective:**

Conventional tumor marker detection technologies are confined to single-index sequential testing, with drawbacks like prolonged turnaround times, high sample consumption, and heavy reliance on large precision instruments. They fail to meet the needs of clinical scenarios such as point-of-care testing (POCT), primary medical institution screening, and emergency rapid assessment. This study aims to develop a multi-index simultaneous quantitative detection system, providing an efficient, convenient, and reliable technical solution for early accurate diagnosis and whole-course dynamic monitoring of tumors.

**Methods:**

Leveraging the core advantages of micro fluidic chips—miniaturization, integration, and low reagent consumption—a detection platform was designed to simultaneously quantify six tumor-associated markers: matrix metalloproteinase 9 (MMP-9), vascular endothelial growth factor A (VEGF-A), soluble neural cadherin (sN-cadherin), osteoprotegerin (OPG), lysyl oxidase (LOX), and angiopoietin 2 (ANG-2). Methodological characterization included linear range verification, limit of detection (LOD) determination, precision evaluation, and specificity tests (cross-reactivity, matrix interference, and background interference). Consistency with clinical gold-standard methods was assessed via correlation analysis, Kappa test, and Bland-Altman analysis. Diagnostic efficacy was evaluated using ROC curve analysis, and detection timeliness was improved by optimizing the “two-reaction and two-washing” core process.

**Results:**

All six markers exhibited excellent analytical performance: the coefficients of determination (R²) of their dose-response curves ranged from 0.9968 to 0.9993, with linear ranges of 0.012–16,000 pg/mL (VEGF-A, OPG, ANG-2) and 0.016–9,600 ng/mL (MMP-9, sN-cadherin, LOX), and limits of detection (LODs) of 0.012–0.019 pg/mL (or equivalent ng/mL units). Precision was outstanding: intra-batch relative standard deviations (RSDs) were 2.51%–5.12% for low-concentration samples and 0.88%–4.14% for high-concentration samples, while inter-batch RSDs, chip repeatability RSDs, and storage stability RSDs were all ≤ 6.76%, meeting the clinical threshold standard of ≤10%. Specificity verification showed that both cross-reactivity rates and interference rates were significant non-specific binding or matrix interference observed. Compared with the gold standard, the coefficient of determination (R²) was > 0.95, the Kappa coefficient was 0.8–1.0 (excellent agreement), and over 90% of sample deviations fell within the 95% confidence interval (CI). The area under the receiver operating characteristic curve (AUC) ranged from 0.9546 to 0.9882, with both detection sensitivity and specificity reaching 93%–98%. The detection system shortened the total detection time to 24 minutes, required only microliter-scale sample consumption, and was 5–8 times faster than enzyme-linked immunosorbent assay (ELISA)/chemiluminescence immunoassay.

**Conclusion:**

The microfluidic chip system integrates high sensitivity, precision, specificity, rapidity, and miniaturization, achieving high equivalence with traditional methods. It breaks through conventional limitations, meeting clinical needs for early screening, dynamic monitoring, and large-scale surveillance, with significant clinical transformation and industrialization prospects. Future optimization will involve multi-center validation, panel expansion, and AI integration to support personalized tumor care.

## 1. Introduction

As the terminal stage of malignant tumor progression, tumor metastasis is the primary cause of death among cancer patients worldwide, accounting for more than 90% of all cancer-related mortalities. This complex pathological process is not an isolated event but a cascade reaction consisting of multiple interconnected key steps, including extracellular matrix (ECM) degradation, angiogenesis, epithelial-mesenchymal transition (EMT), pre-metastatic niche remodeling, and distant organ colonization: cancer cells first secrete proteases to disrupt the surrounding ECM barrier, then undergo EMT to acquire migratory and invasive capabilities, enter the circulatory system under the action of angiogenic factors, and finally colonize and form metastases in the pre-metastatic niche of target organs [1–3]. Therefore, accurate evaluation of tumor invasive and metastatic potential in the early stage of tumor progression not only provides core references for clinicians to formulate personalized treatment plans but also reduces the risk of distant metastasis through early intervention, significantly improving patients’ disease-free survival (DFS) and overall survival (OS). It holds an irreplaceable and crucial significance for enhancing the overall efficacy of cancer treatment and reducing mortality [4].

However, the currently commonly used single tumor marker detection strategy in clinical practice has significant limitations: since tumor metastasis is regulated by the crosstalk of multiple signaling pathways, a single marker can only reflect the pathological changes in one link of the metastatic process and cannot fully cover the molecular characteristics of the entire metastatic cascade, leading to generally low sensitivity and specificity of detection results [5–6]. For example, detecting only vascular endothelial growth factor (VEGF) fails to assess the local invasive ability of cancer cells, while detecting matrix metalloproteinases (MMPs) alone is insufficient to reflect the vascular supply status of metastases [7]. The limitations of single-index detection make it difficult for clinicians to comprehensively and accurately judge the risk of tumor metastasis, thereby affecting the scientificity of treatment decisions. Therefore, developing an efficient, high-throughput, and high-specificity simultaneous detection technology for multiple markers to achieve a panoramic evaluation of tumor metastasis-related molecular events has become an urgent core problem in the fields of laboratory medicine and oncology [8–9].

At present, the commonly used detection methods for tumor markers in clinical practice and scientific research mainly include enzyme-linked immunosorbent assay (ELISA), chemiluminescence immunoassay (CLIA), and liquid chromatography-tandem mass spectrometry (LC-MS/MS) [10]. Among them, ELISA is widely used in primary medical institutions and routine detection scenarios due to its simple operation process, low reagent cost, and no need for complex instruments and equipment. However, this method has obvious drawbacks: traditional ELISA adopts a microplate incubation mode, with a detection cycle as long as 2–3 hours, limited throughput, and inability to meet the demand for simultaneous detection of multiple indicators; meanwhile, its ability to detect trace markers in serum (concentration below ng/mL level) is insufficient, making it difficult to meet the sensitivity requirements for early tumor metastasis evaluation [11–12]. Although CLIA has improved the detection sensitivity to the pg/mL level through chemiluminescence signal amplification technology, it relies on large-scale precision detection instruments (such as automatic chemiluminescence immunoanalyzers), which have high equipment purchase and maintenance costs. Moreover, the operation process requires professional technical personnel, limiting its application in point-of-care testing (POCT), primary medical care, and emergency detection scenarios [13]. Although LC-MS/MS technology has high specificity and multi-component analysis capabilities, it involves complex sample pretreatment, long detection cycles, and high instrument operation thresholds, making it difficult to achieve routine clinical application [14].

In contrast, as a novel analytical technology based on microelectromechanical systems (MEMS), microfluidic chip technology has emerged as an ideal technical carrier for the simultaneous detection of multiple markers, thanks to its core advantages including miniaturized channels (mostly at the micrometer scale), low sample consumption (several microliters to tens of microliters), high reaction efficiency, and strong integration [15–16]. Recent research advances in this field have further expanded its clinical application prospects, such as liquid biopsy instruments for the ultra-fast and label-free detection of circulating tumor cells (CTCs) [17], self-assembled DNA molecular machines enabling rapid, homogeneous, and portable quantitative detection of lung cancer CTCs [18], and multimodal early screening systems integrating machine learning with multiplexed laser-induced graphene immunosensors [19]. All these studies have verified the feasibility of microfluidic technology in high-performance tumor-related detection.

Microfluidic chip technology can integrate multiple experimental steps—including sample pretreatment, antigen-antibody reaction, signal separation, and detection—onto a single chip, significantly shortening the detection cycle and reducing human error [20]. Meanwhile, as an efficient immunoassay technology, magnetic particle immunofluorescence assay uses magnetic particles modified with antibodies on their surface as capture carriers. Under the action of an external magnetic field, it can rapidly separate and enrich target analytes, greatly improving detection specificity. Additionally, combined with fluorescein-labeled detection antibodies and a fluorescence signal reading system, it achieves accurate quantification of target markers by leveraging the high sensitivity of fluorescence signals [21–22].

The integration of microfluidic chip technology and magnetic particle immunofluorescence assay can fully exert their synergistic advantages: the high-throughput and integrated characteristics of microfluidic chips address the efficiency challenge in the simultaneous detection of multiple markers, while the high-specificity capture of magnetic particles and the high sensitivity of fluorescence detection overcome the technical difficulty in detecting trace analytes. Ultimately, this integrated technology enables efficient, rapid, and accurate simultaneous detection of multiple tumor markers in complex biological samples such as serum and plasma [23–25].

It is important to emphasize that despite the significant advantages of the integrated microfluidic-magnetic bead multi-marker detection platform, its clinical translation still faces two key bottlenecks: non-specific binding interference caused by complex clinical sample matrices (e.g., serum proteins, lipids, etc.), and potential weak cross-interference in high-throughput parallel detection. To address these issues, targeted design strategies have been incorporated into the platform development: non-specific adsorption is inhibited through optimized surface blocking protocols and standardized washing procedures, reducing background noise induced by non-specific matrix interactions; for cross-interference control, the inherent specificity of commercial monoclonal antibodies is leveraged, combined with the structural design of the microfluidic platform, to minimize potential signal crosstalk between parallel detection channels. These design principles provide crucial support for the reliable application of the platform in real-world clinical scenarios, with detailed validation data presented in subsequent sections.

To fully cover the key pathological links of the entire tumor metastatic cascade and ensure the scientificity and clinical value of combined detection, this study systematically reviewed the molecular mechanisms of tumor metastasis and clinical research evidence, and selected 6 core markers with clear biological functions to construct a detection panel: ① Matrix Metalloproteinase 9 (MMP-9): As a key member of the MMPs family, it can specifically degrade ECM components such as type Ⅳ collagen, and is a core molecule for cancer cells to break through tissue barriers and initiate local invasion [26]; ② Vascular Endothelial Growth Factor A (VEGF-A): By binding to receptors on the surface of vascular endothelial cells, it induces angiogenesis, providing necessary blood circulation channels for the nutritional supply and distant metastasis of cancer cells [27]; ③ Soluble N-cadherin (sN-cadherin): A core marker of EMT transition. Cancer cells acquire mesenchymal cell phenotypes by downregulating E-cadherin and upregulating N-cadherin expression, thereby enhancing migratory and invasive capabilities. Its soluble fragments can stably exist in serum, directly reflecting the degree of EMT transition [28]; ④ Osteoprotegerin (OPG): By regulating osteoclast activity and the bone metabolic microenvironment, it specifically mediates the colonization and growth of tumor cells in bone tissue, and is a key marker for early warning of tumor bone metastasis [29]; ⑤ Lysyl Oxidase (LOX): It remodels the ECM by catalyzing the cross-linking reaction of collagen and elastin, constructing a pre-metastatic niche suitable for cancer cell colonization. Its high expression is significantly associated with the risk of distant tumor metastasis [30]; ⑥ Angiopoietin-2 (ANG-2): It synergizes with VEGF-A to regulate the maturation and stability of new blood vessels, further strengthening the tumor angiogenesis network and providing continuous support for the growth of metastases [31]. These 6 markers correspond to the key steps of tumor metastasis (“local invasion - angiogenesis - cell migration - microenvironment remodeling - distant colonization”), forming a complete molecular detection chain. Their combined detection can comprehensively and systematically reflect the overall potential of tumor invasion and metastasis, providing more reliable molecular evidence for clinical evaluation.

Based on the above research background and gaps, this study aims to construct a microfluidic chip-based magnetic particle immunofluorescence detection platform for the simultaneous quantitative detection of MMP-9, VEGF-A, sN-cadherin, OPG, LOX, and ANG-2. The specific research contents include: optimizing the channel structure and reaction area design of the microfluidic chip to improve the compatibility of multi-channel parallel detection; optimizing the magnetic particle-antibody conjugation conditions and immune reaction parameters; systematically evaluating the core performance indicators of the detection platform, including detection sensitivity (expressed as limit of detection, LOD), specificity (anti-cross-reactivity), linear detection range, and precision; and collecting serum samples from clinical tumor patients and healthy controls to verify the practical clinical application potential of the platform. It is expected that the integrated detection platform constructed in this study can achieve rapid (detection time ≤ 1 hour), micro-volume (sample volume ≤ 50 μL), and high-throughput simultaneous detection of 6 tumor invasion and metastasis markers. It will provide a novel and accurate detection tool for the early warning, risk stratification, efficacy monitoring, and prognosis evaluation of tumor invasion and metastasis, thereby offering technical support for the formulation of personalized cancer treatment strategies and promoting the development of precision oncology.

## 2. Materials and methods

### 2.1. Study subjects

This study has been approved by the Medical Ethics Committee (Approval No.: MEC-XSMC-KT-20250910–001). All clinical serum samples were collected in strict compliance with medical ethics guidelines and with written informed consent obtained from all patients. A total of 360 baseline clinical serum samples were enrolled in the study for the detection of six biomarkers, including matrix metalloproteinase-9 (MMP-9), vascular endothelial growth factor-A (VEGF-A), soluble N-cadherin (sN-cadherin), osteoprotegerin (OPG), lysyl oxidase (LOX), and angiopoietin-2 (ANG-2), with 60 samples allocated to each biomarker. These baseline samples were collected from the First Affiliated Hospital of Xinjiang Second Medical College and Karamay City People’s Hospital between September 12 and December 12, 2025. The donors of the samples were patients with malignant tumors, involving 7 types of diseases such as lung cancer, colorectal cancer, gastric cancer, breast cancer, ovarian cancer, pancreatic cancer, and prostate cancer. The disease stages coveredⅠ-Ⅳ, with a higher proportion of patients in stagesⅡ-Ⅳ.

During sample selection, newly diagnosed patients were prioritized, requiring no prior receipt of chemoradiotherapy, surgical treatment, or targeted therapy. Among them, patients who had received anti-angiogenic therapy were excluded from the VEGF-A and ANG-2 detection samples, while patients who had undergone bone-protective therapy were excluded from the OPG detection samples. All samples were collected via fasting venous blood sampling, with a blood volume of 5 mL for VEGF-A and OPG detection. After collection, the blood was allowed to clot at room temperature for 30 minutes, followed by centrifugation at 3000 r/min for 10 minutes to separate the serum. All serum samples were stored at −20°C in a refrigerator, with repeated freeze-thaw cycles strictly avoided throughout the process. The exclusion criteria for samples were as follows: patients with severe benign diseases or autoimmune diseases; pregnant women were additionally excluded from VEGF-A and ANG-2 detection samples; patients with primary bone diseases were excluded from OPG detection samples; and patients with congenital heart disease or severe vascular diseases were excluded from ANG-2 detection samples.

To further verify the clinical practical value of the detection method, an additional 36 supplementary clinical samples were included in the study. These supplementary samples were collected from the aforementioned two medical institutions between May 1 and May 14, 2026. They were divided into three categories: 12 fully negative samples, derived from healthy individuals without a history of malignant tumors or severe benign diseases, with normal results from laboratory and imaging examinations; 12 single-positive samples, with 2 samples corresponding to each biomarker, all meeting the inclusion criteria of the baseline samples and testing positive only for the target biomarker; and 12 multiple positive samples, including 6 biomarker combination patterns (MMP-9 + VEGF-A, VEGF-A + ANG-2, MMP-9 + VEGF-A + ANG-2, sN-cadherin+LOX, OPG + LOX, sN-cadherin+OPG + LOX), with 2 samples for each combination. All multiple positive samples met the inclusion criteria of the baseline samples and tested positive for all biomarkers in the target combination. The collection process, storage conditions, and exclusion criteria for the supplementary samples were completely consistent with those of the 360 baseline detection samples.

### 2.2. Instruments and reagents

#### 2.2.1. Instruments.

HZZ-V3000 chip laser cutting machine (Huazhizun Optoelectronic Technology Co., Ltd., China), X350 microfluidic chip vacuum heat press (Xingweikong Biotechnology Co., Ltd., China), Olympus OLS5100 laser confocal fluorescence microscope (Olympus Corporation, Japan), Multiskan FC automated enzyme-linked immunosorbent assay reader (Thermo Fisher Scientific, USA), and H2D 3D printer (Bambu Lab Technology Co., Ltd., China), BS-830 Automatic Biochemical Analyzer (Mindray Biomedical Electronics Co., Ltd., China).

#### 2.2.2. Reagents.

Anti-human matrix metalloproteinase-9 (MMP-9) monoclonal antibody (1 mg/mL) was purchased from R&D Systems, USA; human MMP-9 ELISA kit (96-well plate/kit) was acquired from Cusabio Biotech Co., Ltd., Wuhan, China. Anti-human vascular endothelial growth factor-A (VEGF-A) monoclonal antibody (1 mg/mL) was obtained from Thermo Fisher Scientific, USA; human VEGF-A ELISA kit (96-well plate/kit) was purchased from MLBIO Biotechnology Co., Ltd., Shanghai, China. Anti-human soluble N-cadherin (sN-cadherin) monoclonal antibody (1 mg/mL) was purchased from Abcam, UK; human sN-cadherin ELISA kit (96-well plate/kit) was obtained from Shenzhen Xinbosheng Biotechnology Co., Ltd., China. Anti-human osteoprotegerin (OPG) monoclonal antibody (1 mg/mL) was acquired from Merck, USA; human OPG ELISA kit (96-well plate/kit) was purchased from Cusabio Biotech Co., Ltd., Wuhan, China. Anti-human lysyl oxidase (LOX) monoclonal antibody (1 mg/mL) was purchased from R&D Systems, USA; human LOX ELISA kit (96-well plate/kit) was obtained from MLBIO Biotechnology Co., Ltd., Shanghai, China. Anti-human angiopoietin-2 (ANG-2) monoclonal antibody (1 mg/mL) was acquired from Abcam, UK; human ANG-2 ELISA kit (96-well plate/kit) was purchased from Shenzhen Xinbosheng Biotechnology Co., Ltd., China. Carboxyl-modified magnetic beads (10 mg/mL) and carboxyl-modified fluorescent microspheres (10 mg/mL) were purchased from Zhongke Leiming Co., Ltd., China. Polymethyl methacrylate (PMMA) chip substrates were obtained from Trinseo, USA. Other analytical grade reagents (e.g., phosphate-buffered saline, blocking buffer, washing solution) were purchased from Sinopharm Chemical Reagent Co., Ltd., China.

### 2.3. 2D/3D design and fabrication process of microfluidic chips

**Fig 1:**
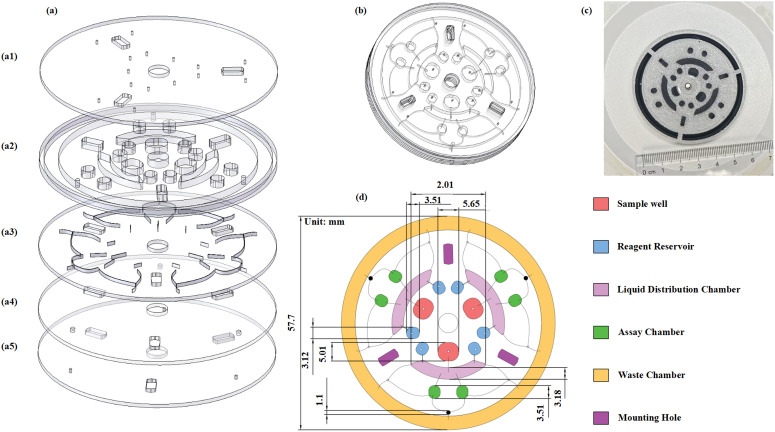
Schematic diagrams of the chip structure and optical photograph of the fabricated chip. **(a)** Exploded schematic diagram of the three-dimensional (3D) chip structure, (a1) top layer of the chip; (a2) chamber layer of the chip, (a3) microchannel layer of the chip, (a4) valve layer of the chip, (a5) substrate layer of the chip, **(b)** Overall schematic diagram of the 3D chip structure, **(c)** Optical photograph of the as-fabricated chip, **(d)** Two-dimensional (2D) schematic diagram of the chip structure.

In this study, the structural design and optimization of the microfluidic chip were firstly completed using 2D and 3D design software. Subsequently, the chip was fabricated via laser cutting and 3D printing techniques. Finally, the bonding and assembly of the chip were realized through vacuum hot-pressing technology, with the detailed process illustrated in Fig 1.

The exploded view of the chip’s 3D structure (Fig 1a) consists of five layers: the top layer (Fig 1a1) for air pressure balancing; the chamber layer (Fig 1a2) for fluid storage; the channel layer (Fig 1a3) for fluid transportation; the valve layer (Fig 1a4) for precise control of fluid movement; and the base layer (Fig 1a5) for chip packaging. The 3D structure diagram of the chip (Fig 1b) clearly shows the overall configuration, the fabricated physical image (Fig 1c) intuitively presents the actual morphology of the chip after fabrication, and the 2D structure schematic (Fig 1d) explicitly labels the functional divisions of each chamber.

This integrated processing strategy aims to realize the precision forming and efficient fabrication of microstructures for the microfluidic chip, guarantee the sealing property, structural stability and mechanical adaptability of the chip microchannels, and enable it to perfectly match the requirements of fluid transport, immune reaction and signal capture in the magnetic particle immunofluorescence detection system. It provides a core carrier with reliable structure and stable performance for the construction of a multi-index combined rapid detection platform, and lays a solid process foundation for improving the accuracy and repeatability of detection results.

### 2.4. Immobilization process of antigens and antibodies on carboxyl-functionalized microspheres

This study achieved the immobilization of antigens and antibodies on carboxyl-modified microspheres via chemical conjugation, with the detailed procedure as follows: First, carboxyl-modified microspheres were washed with 2-(N-morpholino)ethanesulfonic acid (MES) buffer to thoroughly remove residual impurities. Second, 50 mM 1-ethyl-3-(3-dimethylaminopropyl)carbodiimide (EDC) was added for 15-minute activation, converting carboxyl groups into highly reactive intermediates. Third, after activation, specific antigens or antibodies targeting six tumor markers (MMP-9, VEGF-A, sN-cadherin, OPG, LOX, ANG-2) were separately added, followed by 16-hour incubation at room temperature to achieve covalent binding. Fourth, post-reaction, 5% bovine serum albumin (BSA) solution was used for 1-hour blocking. Finally, the microspheres were washed three times with phosphate-buffered saline (PBS) containing 0.1% Tween-20, yielding carboxyl-modified microspheres conjugated with antigens or antibodies.

To address potential weak cross-interference and non-specific binding during high-throughput simultaneous detection of six markers in a single reaction system with complex clinical sample matrices, multiple targeted designs were integrated into the chemical conjugation process to enhance detection specificity: 1) EDC-mediated covalent conjugation enabled oriented and efficient immobilization of antigens/antibodies. This method forms stable amide bonds via specific condensation between carboxyl and amino groups, effectively preventing the shedding and inactivation of biorecognition molecules, ensuring correct exposure of their antigen-binding sites or epitopes, maintaining high immunological activity, and reducing non-specific binding risk at the molecular mechanism level; 2) The blocking system was optimized: in addition to BSA blocking unreacted active carboxyl sites, 0.05% Tween-20 was added to the blocking buffer to reduce hydrophobic interaction-mediated non-specific adsorption. Meanwhile, blocking time was extended to 1 hour with shaking incubation (100 rpm) to ensure full binding between blocking agent and active sites, maximizing the blocking of non-specific binding between non-target molecules and microsphere surfaces and minimizing false-positive signals; 3) Immune reagents were strictly screened and their ratios optimized: all selected antibodies were validated high-specificity monoclonal antibodies, and their immobilization concentrations were precisely controlled during conjugation. This ensures that immunomagnetic microsphere probes for different markers maintain specific binding capabilities in the same reaction system, avoiding cross-reaction or competitive binding between antibodies.

After the above process optimization, the final immunomagnetic microsphere probes exhibit high binding activity, strong stability, and excellent specificity. They can accurately recognize six target markers in complex clinical serum samples (containing interfering components such as proteins, lipids, and metabolites), effectively inhibiting weak cross-interference and non-specific binding. As the core immune recognition component of the magnetic particle immunofluorescence detection system, these probes lay a critical technical foundation for ensuring detection sensitivity, specificity, and the accuracy and reliability of results.

### 2.5. Method for optimizing the mixing ratio of additives

To optimize the conjugation efficiency of antibodies/antigens onto carboxyl-modified microspheres and determine the optimal reaction conditions, five experimental groups with distinct mass ratios of microspheres to antibodies/antigens (10:1, 25:1, 50:1, 75:1, and 100:1) were designed in this study. All groups were incubated with stirring for 3 h in a dark environment. Following blocking with bovine serum albumin (BSA) solution, the concentration of free antibodies in the supernatant was quantitatively determined using ultraviolet spectrophotometry. The conjugation efficiency was calculated according to the following formula:Conjugation efficiency (%) = (Initial total mass of antibodies – Mass of free antibodies in the supernatant)/ Initial total mass of antibodies × 100%.Each experiment was performed in triplicate, and the mass ratio corresponding to the maximum conjugation efficiency was adopted as the final optimized reaction parameter.

To identify the optimal binding conditions between the test samples and carboxyl-modified fluorescent microspheres conjugated with anti-human IgG antibodies, the same positive sample was serially diluted with a 2% BSA solution (containing 0.05% Tween-20) prepared in 0.01 mol/L phosphate-buffered saline (PBS, pH 7.4) at four gradient dilutions (1:50, 1:100, 1:200, and 1:400). Samples at each dilution were separately incubated with 1 μg, 5 μg, and 10 μg of activated carboxyl-modified fluorescent microspheres. After incubation, the fluorescence signal value and background signal value of each group were detected, and the ratio of fluorescence intensity to background fluorescence was calculated. The experimental condition corresponding to the maximum ratio was selected as the optimal parameter. The entire experiment was repeated three times to ensure the reliability and repeatability of the results.

The core objective of the two sets of parameter optimization experiments designed in this study is to systematically screen the key reaction conditions suitable for the detection system. The first experiment focuses on the conjugation process of antibodies with carboxyl-modified microspheres. By quantifying the conjugation efficiency at different mass ratios, it achieves the efficient immobilization of biorecognition molecules on the microsphere surface, which not only avoids the shortage of effective binding sites caused by insufficient molecule dosage but also reduces resource waste and non-specific interference resulting from excessive molecules. The second experiment concentrates on the interaction between the test sample and fluorescent microspheres. By regulating the sample dilution gradient and microsphere dosage, it optimizes the ratio of fluorescence signal to background noise, thereby enhancing the response sensitivity and recognition specificity of the detection system for target analytes. The parallel replicates and standardized operating procedures adopted in the experiments ensure the statistical reliability and practical application stability of the screened parameters. Ultimately, this study aims to establish an immunofluorescence detection system with controllable reaction conditions and excellent performance, providing key parameter support and technical backing for the accurate implementation of subsequent multi-index combined detection.

### 2.6. Hydrodynamic simulation test method

The microfluidic chip designed in this study adopts a multi-channel disc-shaped structure and achieves uniform liquid splitting through its symmetric configuration. To verify the applicability of this chip in fluid manipulation, this study performed channel pressure tests using the hydrodynamic simulation software Comsol Multiphysics 6.3, and simultaneously measured the wall resolution to ensure the authenticity and reliability of the simulation results. The parameters for the hydrodynamic test were set as follows: the serum density was set to 1020 kg/m³ according to the research by Craft et al. [32]; the serum viscosity was determined as 1.2 mPa·s based on the data from Rosenson et al. [33]; the surface tension was set to 48 mN/m in accordance with the findings of Rosina et al. [34]; and the fluid flow mode was set as laminar flow referring to the study by Duffy et al. [35]. The theoretical support for the flow velocity calculation formula is derived from the organic combination of the Poiseuille flow principle and the radial pressure gradient induced by centrifugal force.

The key to centrifugal force-driven motion lies in the centrifugal acceleration generated by rotation.


$$\begin{array}{c} \alpha =\omega^{\text{2}}r \end{array}$$ (1)

Herein, ω represents the angular velocity (unit: radian per second), and the angular velocities set for this chip are 4 rad/s, 8 rad/s, 12 rad/s, and 16 rad/s in sequence; r denotes the rotational radius (unit: meter), and the rotational radii corresponding to the above four angular velocities are 0.08 m, 0.012 m, 0.016 m, and 0.024 m, respectively.

All fluid channels of this chip have a rectangular cross-section, and the calculation formula for the liquid flow velocity in such channels is as follows:


$$\begin{array}{c} \upsilon =\frac{\rho h^{\text{2}}\alpha }{\text{12}\mu } \end{array}$$ (2)

Herein, ρ denotes the fluid density (unit: kg/m³), which is a known parameter; h represents the channel height (unit: m) with a value of 0.00024 m; α stands for the centrifugal acceleration (unit: m/s²), which is obtained through calculation using Formula 1; andμ refers to the dynamic viscosity of the fluid (unit: Pa·s), which is also a known parameter.

After calculating the liquid flow velocity in the chip via the aforementioned formula, hydrodynamic simulation tests are conducted in accordance with the above parameters.

The experiments in this section are conducted to pre-verify the rationality of the chip’s fluid flow design and avoid the trial-and-error costs associated with the fabrication of physical microfluidic chips.

### 2.7. Key experimental parameters, operational specifications, and result determination criteria for the multi-channel disc-shaped magnetic immunofluorescence microfluidic chip

The disc-shaped microfluidic chip selected in this study had an overall specification of 5.77 cm in diameter and 2.4 mm in thickness, with integrated microchannels of 120 μm in width and 240 μm in depth. To achieve the ordered transfer of fluids among various functional regions of the chip, a gradient centrifugation protocol was established: the centrifugation speed was set at 80 r/min when transferring fluids from the sample chamber and fluorescent microsphere chamber to the distribution pool; adjusted to 120 r/min during the transfer of fluids from the distribution pool to the detection chamber; and fixed at 160 r/min for the transfer of reacted fluids from the detection chamber to the waste pool. The duration of each centrifugation stage was consistently 36 seconds. In the immunoreaction step, the first incubation was set at 37°C for 7 minutes, and the second incubation was set at 37°C for 9 minutes.

**Fig 2:**
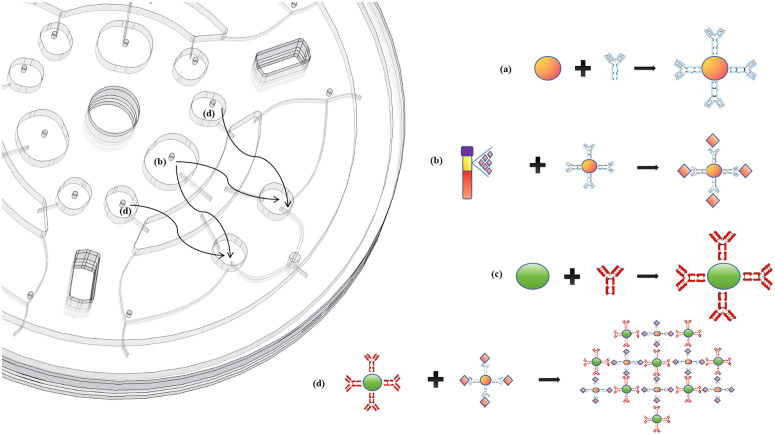
Schematic diagram of the chip detection process. **(a)** Antibodies are pre-coated onto magnetic beads, **(b)** After the sample is added, the antigen in the sample binds to the magnetic bead-antibody complex, **(c)** Antibodies are pre-coated onto fluorescent microspheres, **(d)** The antigen-antibody-magnetic bead complex binds to the antibody-fluorescent microsphere complex.

The specific operational procedure of the chip is as follows: First, the test sample is added to the chip and subjected to centrifugation. The sample then enters the detection chamber through the liquid separation pool (antibody-coated magnetic beads targeting the target tumor markers are preloaded in the chamber, see Fig 2a). After incubation and washing, the tumor markers in the sample specifically bind to the antibody-coated magnetic beads to form antigen-antibody-magnetic bead complexes (see Fig 2b). Subsequently, centrifugation is performed again under magnetically assisted conditions to transfer unbound excess components to the waste liquid pool. Next, the antibody-coated fluorescent microsphere solution (as shown in Fig 2c) is added, and the solution enters the detection chamber through the liquid separation pool. After another round of incubation and washing, the formed complexes specifically bind to the secondary antibody-fluorescent microspheres, further constructing magnetic bead-antibody-antigen-antibody-fluorescent microsphere complexes (Fig 2d). All excess waste liquid generated during the entire operation is collected into the waste liquid pool by centrifugation. Finally, fluorescence signal detection is performed in the detection chamber, and the quantitative detection results of the target tumor markers in the serum samples can be obtained after data collation and analysis. The detailed operational procedure is shown in Fig 2.

The criteria for result judgment are set as follows: Taking the detection results of the clinically conventional Enzyme-Linked Immunosorbent Assay (ELISA) as the reference standard, the detection data of 60 positive samples and 60 negative samples were selected respectively for the six indicators: anti-human matrix metalloproteinase-9 (MMP-9), anti-human vascular endothelial growth factor-A (VEGF-A), anti-human soluble N-cadherin (sN-cadherin), anti-human osteoprotegerin (OPG), anti-human lysyl oxidase (LOX), and anti-human angiopoietin-2 (ANG-2). The optimal cut-off value for each indicator detected by this chip was determined through Receiver Operating Characteristic (ROC) curve analysis. Samples with detection signal values higher than the optimal cut-off value were judged as positive, and those lower than the cut-off value were judged as negative.

The core objective of the established chip operating procedure and result judgment criteria in this study is to develop a standardized and integrated quantitative detection system for tumor markers. Leveraging the modular structural design of the chip’s internal liquid separation chamber, detection chamber and waste chamber, combined with centrifugal driving technology and magnetic-assisted separation strategy, the key steps including sample injection, specific binding, washing and impurity removal, signal amplification and waste collection are integrated into an efficient one-stop workflow. This not only greatly simplifies the operational complexity and shortens the overall detection time, but also ensures the specific recognition efficiency of tumor markers with the double antibodies (antibody-coated magnetic beads and antibody-coated fluorescent microspheres) through precise hydrodynamic control and targeted immune binding reaction, thereby reducing the detection interference caused by non-specific binding.

Using the clinically widely recognized ELISA detection results as the reference standard, the optimal cut-off value of each target indicator (six indicators including MMP-9 and VEGF-A) is determined via receiver operating characteristic (ROC) curve analysis, which guarantees the consistency of the chip detection results with clinical standards and the accuracy of judgment, and enhances the clinical guiding significance of positive/negative classification. Ultimately, this design aims to achieve rapid, accurate and highly specific quantitative detection of the above six tumor markers, providing reliable technical support for early tumor screening and dynamic disease monitoring, and laying a standardized foundation for the clinical transformation of multi-index combined detection technology.

### 2.8. Experimental methods for performance evaluation of microfluidic chips

To establish the dose-response standard curves for six tumor markers, serially diluted standard samples of each indicator were prepared in this study. The diluent matched with the corresponding ELISA kits was used as the dilution medium to dilute the standard substances provided by the kits. The initial concentration of matrix metalloproteinase-9 (MMP-9) was 10000 ng/mL, with concentration gradients of 0, 600, 1200, 2400, 4800 and 9600 ng/mL. The initial concentration of vascular endothelial growth factor-A (VEGF-A) was 5000 pg/mL, with gradients of 0, 300, 600, 1200, 2400 and 4800 pg/mL. Soluble N-cadherin (sN-cadherin) had an initial concentration of 1000 ng/mL, with gradients of 0, 60, 120, 240, 480 and 960 ng/mL. Both osteoprotegerin (OPG) and angiopoietin-2 (ANG-2) had an initial concentration of 20000 pg/mL, with gradients of 0, 1000, 2000, 4000, 8000 and 16000 pg/mL. Lysyl oxidase (LOX) was prepared at an initial concentration of 2000 ng/mL, with gradients of 0, 100, 200, 400, 800 and 1600 ng/mL. Pure diluent was adopted as the blank control at the zero-concentration level. All diluted standard samples were detected using self-developed microfluidic chips, and three parallel replicates were set for each concentration to record the corresponding fluorescence signal intensity accurately. Taking standard substance concentration as the abscissa and fluorescence signal intensity as the ordinate, the sigmoidal dose-response curves were fitted via the variable-slope four-parameter logistic model widely applied in immunoassays. To optimize the detection performance at low concentration ranges and improve the accuracy of limit of detection, additional low-concentration samples were further prepared and determined. Specifically, low-concentration gradients ranging from 0.02 to 0.64 ng/mL were configured for MMP-9, sN-cadherin and LOX, while gradients from 0.02 to 0.64 pg/mL were set for VEGF-A, OPG and ANG-2. All low-concentration samples were also detected in triplicate with fluorescence signals recorded for subsequent establishment of complete dose-response curves.

Determination of the Limit of Detection (LOD): Ten parallel tests were performed on the 0-concentration standard (blank control). The fluorescence intensity data were collected to calculate the mean value ($\bar{x}$) and standard deviation (SD). The fluorescence signal value corresponding to $\bar{x}$ + 2SD was substituted into the established dose-response curve equation, and the calculated concentration value was defined as the LOD of the chip.

Accuracy Validation:Three types of control samples were set: positive control, negative control, and blank control. The positive and negative controls were commercial ELISA kit standard products, and the blank control was phosphate-buffered saline (PBS, pH 7.4). The three types of control samples were detected by the chip, and the reaction results under bright and dark fields were observed by fluorescence microscopy, with three parallel replicates for each sample. The detection accuracy of the chip was evaluated based on the consistency between the detection results and the control standards.

For the six target biomarkers, namely matrix metalloproteinase-9 (MMP-9), vascular endothelial growth factor-A (VEGF-A), soluble N-cadherin (sN-cadherin), osteoprotegerin (OPG), lysyl oxidase (LOX), and angiopoietin-2 (ANG-2), four sets of high- and low-concentration paired samples (each set containing one high-value sample and one low-value sample) were individually prepared for each biomarker. The concentrations of all samples were double-confirmed by enzyme-linked immunosorbent assay (ELISA) and chemiluminescence immunoassay (CLIA), and their concentration ranges covered the common levels of the corresponding biomarkers in clinical specimens. It is worth noting that the aforementioned four sets of paired samples were independently designed, corresponding to four methodological validation indicators: intra-batch precision, inter-batch precision, chip reproducibility, and long-term stability. For intra-batch precision validation, microfluidic chips and supporting reagents from the same production batch were used to perform simultaneous quantitative detection of the dedicated high- and low-concentration paired samples for each biomarker, with 10 replicate wells set for each sample. The mean value, standard deviation (SD), and relative standard deviation (RSD) of the detection results for each biomarker were calculated, and intra-batch precision was evaluated based on the degree of result dispersion reflected by the RSD value; referring to the relevant standards for clinical laboratory quality control, the acceptable threshold for intra-batch precision was set at RSD < 10%. For inter-batch precision validation, to clarify the potential impact of batch differences in chips and reagents on the consistency of detection results, three batches of microfluidic chips were prepared at 1-week intervals, and the dedicated paired samples for each biomarker were tested in parallel using chips from each batch, with 5 replicate wells set for each sample in each batch. The mean detection value of each biomarker in each batch was first calculated, and then the inter-batch RSD was obtained by comparing the mean values of the three batches; the acceptable standard was set at RSD < 15% to ensure that batch differences during chip prepay of the detection system. Chip reproducibility validation aimed to assess the consistency of detection performance among different individual chips from the same production batch. Fifteen chips were randomly selected from the same batch, and each chip completed one round of simultaneous quantitative detection of the dedicated high- and low-concentration paired samples for each biomarker. The RSD value of the detection results from the 15 chips was statistically analyzed, and the acceptable standard was set at RSD < 10% to further verify the uniformity of the microfluidic chip manufacturing process and ensure the reliability and consistency of detection results from different individual chips. Long-term stability validation focused on the storage stability of the chips; the microfluidic chips were stored at −20°C for 1 month and 3 months respectively before detection, with freshly prepared chips (storage duration of 0 months) used as control samples. At each storage time point, 3 chips were randomly selected to detect the dedicated paired samples for each biomarker, and the RSD of the detection results for each biomarker at different storage time points was required to be < 15%, thereby confirming that the chips could maintain stable detection performance under the specified storage conditions.ration and reagent formulation would not interfere with the overall stability.

Specificity verification was performed using a combination of cross-reactivity assays, interference tests, and negative control experiments to systematically evaluate the detection specificity of the microfluidic chip. In the cross-reactivity assays, high-concentration non-target antigen solutions corresponding to six target detection indices (MMP-9, VEGF-A, sN-cadherin, OPG, LOX, and ANG-2) were prepared using purified antigen positive controls compatible with ELISA kits as raw materials. Subsequently, the constructed microfluidic chip was used to determine each of the six detection items individually, with five parallel replicate wells set for each non-target antigen. Taking the specific binding signal of the target antigen as the reference standard, the cross-reactivity rate (CR) was calculated according to the formula: CR (%) = (Detection signal value of non-target antigen/ Detection signal value of target antigen) × 100%.

For the interference tests, five clinical samples each of hemolyzed, lipemic, and icteric sera were collected. The concentration ranges of key interferents were determined and confirmed using an automatic biochemical analyzer: hemoglobin (Hb) concentration in hemolyzed serum was 10–50 g/L, triglyceride (TG) concentration in lipemic serum was 5.6–22.6 mmol/L, and total bilirubin (TBIL) concentration in icteric serum was 34.2–171 μmol/L. Each type of interfering serum sample was mixed with the target antigen standard at a volume ratio of 1:1, and the mixture was detected using the microfluidic chip with five parallel replicates set for each sample. Meanwhile, target antigen standards without interferents were used as blank controls, and the interference rate (IR) was calculated following the formula: IR (%) = |(Detection signal value of interference group – Detection signal value of control group)/ Detection signal value of control group| × 100%.

In the negative control experiments, five healthy human serum samples confirmed negative for all six target indices by clinical testing and five PBS buffer samples were selected. Each sample was detected individually using the microfluidic chip with five parallel replicates, and the background signal values of each detection were recorded synchronously. Comprehensive assessment of the non-specific binding degree between the chip and non-target antigens as well as interfering substances was conducted based on the results of the above three types of experiments, thereby systematically verifying the detection specificity of the microfluidic chip.

In this study, a series of systematic methodological validations were conducted to comprehensively evaluate the core detection performance and clinical application potential of the fabricated microfluidic chip. Specifically, the calibration of reference materials and dose-response curve validation aimed to establish a standardized quantitative correlation between the concentrations of target tumor markers and fluorescence signals, providing a calibration standard for accurate quantitative detection. The determination of the limit of detection (LOD) focused on the chip’s responsiveness to low-concentration targets, defining its applicable scope for the clinical screening of low-level tumor markers. Using clinically recognized ELISA standards as references, accuracy validation guaranteed the clinical reliability and comparability of the chip’s detection results through consistency comparison. Precision validation quantified the repeatability and stability of the detection system via repeated tests, reducing the interference of random errors on the detection results. Specificity validation ensured the chip’s highly specific recognition efficiency for target antigens from three key dimensions: cross-reactivity avoidance, matrix interference resistance, and background signal control. These validation experiments fully cover the core technical indicators for the methodological evaluation of clinical detection, and ultimately provide comprehensive and sufficient experimental support for the scientificity, result reliability, and clinical transformation feasibility of the microfluidic chip in the combined detection of multiple tumor markers.

### 2.9. Statistical processing and analysis

Data processing and statistical analysis in this study were performed using Microsoft Excel and GraphPad Prism 10 software. In accordance with the pre-established sample inclusion criteria, this study conducted detections on six types of serum tumor markers, specifically including anti-human matrix metalloproteinase-9 (MMP-9), anti-human vascular endothelial growth factor-A (VEGF-A), anti-human soluble N-cadherin (sN-cadherin), anti-human osteoprotegerin (OPG), anti-human lysyl oxidase (LOX), and anti-human angiopoietin-2 (ANG-2).

At the initial stage of the study, to construct a basic detection dataset, 60 positive serum samples and 60 negative serum samples were enrolled for each marker, totaling 720 samples in this part. To further systematically verify the specificity and accuracy of the detection method, an additional 36 clinical samples were supplemented in this study and divided into three subgroups based on the positive characteristics of the markers: ① 12 fully negative samples, where the detection results of all six target markers were negative; ② 12 single-positive subgroup samples, with 2 exclusive samples corresponding to each marker—only the target marker was positive, while the other five markers were negative; ③ 12 multiple-positive subgroup samples, covering five positive combination patterns: 2 cases of MMP-9 + VEGF-A double positive, 2 cases of VEGF-A + ANG-2 double positive, 2 cases of MMP-9 + VEGF-A + ANG-2 triple positive, 2 cases of sN-cadherin+LOX double positive, 2 cases of OPG + LOX double positive, and 2 cases of sN-cadherin+OPG + LOX triple positive. For all multiple-positive samples, only the target combination of markers was positive, and the detection results of the remaining markers were negative.

Combining the initial dataset and the supplementary validation dataset, the total number of detected samples ultimately included in this study was 756.

Two detection methods were employed for parallel testing of all samples in this study: the antibody detection technology based on a microfluidic chip independently established herein, and the clinically widely used enzyme-linked immunosorbent assay (ELISA) antibody detection method. To reduce the impact of systematic errors and random errors on the results, each sample was subjected to three replicate determinations using both detection methods. The raw detection data were systematically organized using Microsoft Excel software and then imported into GraphPad Prism 10 software for subsequent statistical analysis.

For the 720 samples (including positive and negative samples for each marker), consistency verification was performed between the microfluidic chip and ELISA method, with specific analytical approaches as follows: ① Correlation analysis: A correlation scatter plot was generated with the microfluidic chip detection results as the abscissa and the ELISA detection results as the ordinate. The correlation coefficient (R²) was used to evaluate the correlation between the two methods, and an R² ≥ 0.95 indicated a good correlation; ② Kappa test: This was used to quantify the consistency of detection results between the two methods, and a Kappa value ranging from 0.8 to 1.0 suggested excellent consistency; ③ Difference-based Bland-Altman analysis: The differences between the detection results of the two methods were calculated. If more than 90% of the sample difference data fell within the 95% confidence interval, it further confirmed that the detection results of the two methods had good consistency.

To more comprehensively verify the detection performance of the microfluidic chip, dual consistency evaluations were conducted between the microfluidic chip and two reference methods (ELISA and chemiluminescence immunoassay, CLIA) for the additional 36 supplementary clinical samples (covering fully negative, single-positive, and multiple-positive subgroups). Both control combinations were analyzed for correlation using correlation scatter plots (an R² ≥ 0.95 was determined as good correlation), and ratio-based Bland-Altman analysis was synchronously adopted to verify consistency, thereby comprehensively ensuring the reliability and clinical applicability of the microfluidic chip detection results.

## 3. Results and discussion

### 3.1. Experimental results of additive ratio optimization

**Fig 3:**
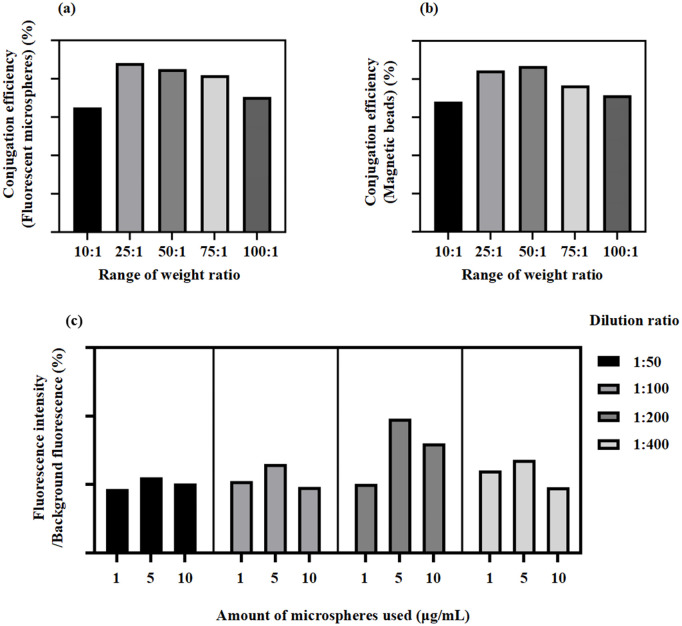
Experimental results of ratio optimization. **(a)** Optimization results for the ratio of antibody-conjugated fluorescent microspheres, **(b)** Optimization results for the ratio of antigen-conjugated magnetic beads, **(c)** Optimization results for sample dilution ratio and microsphere dosage.

Figs 3(a) and 3(b) present the optimization data of the mass ratios between antibody-conjugated magnetic beads and antibody-conjugated fluorescent microspheres, while Fig 3(c) shows the screening results of clinical sample dilution ratios and microsphere dosages. Experimental verification demonstrated that the multi-index combined detection system achieved the optimal immune conjugation efficiency when the mass ratio of antibody-conjugated fluorescent microspheres was 25:1 and that of antigen-conjugated magnetic beads was 50:1; the strongest specific fluorescent signals for the six target tumor markers were obtained when the clinical sample dilution ratio was 1:200 and the microsphere dosage was controlled at 5 μg/mL.

These optimization results determined the core reaction conditions of the six-marker combined detection system. This ratio system effectively balanced the utilization efficiency of immune binding sites and the steric hindrance effect, thereby significantly improving the immune conjugation efficiency. Notably, the optimized surface blocking protocol (5% bovine serum albumin, BSA) and standardized washing process (three washes with phosphate-buffered saline (PBS) containing 0.1% Tween-20) integrated into the reaction system played a key role in inhibiting non-specific binding. Combined with the optimal 1:200 sample dilution ratio, these measures collectively reduced non-specific interference caused by complex serum matrices (such as serum proteins and lipids). Regarding cross-interference control, the inherent specificity of commercial monoclonal antibodies, coupled with the structural design of the microfluidic platform (e.g., isolated parallel channels to avoid physical crosstalk), can minimize potential signal interference between the six markers.

In summary, the optimized parameter system (including immune reagent ratios, sample dilution ratios, and microsphere dosages) not only balances conjugation efficiency and steric hindrance but also, through the synergistic effect of surface blocking, standardized washing, and microfluidic structural design, effectively mitigates non-specific binding interference from complex matrices and potential weak cross-interference in parallel detection. These results directly validate the feasibility of the targeted design strategies proposed in the Introduction, ensuring the platform’s favorable multi-index adaptability, detection stability, and reliability in real-world clinical scenarios. This provides core support for the standardized construction and clinical translation of the detection system.

### 3.2. Results of Hydrodynamic Analysis

**Fig 4:**
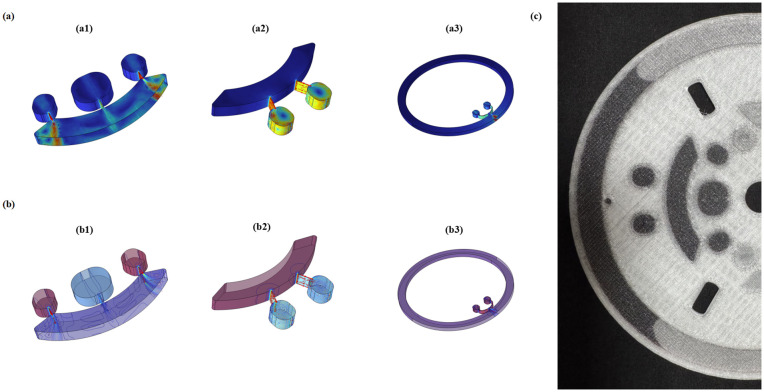
Hydrodynamic tests of the chip and schematic diagram of the actual liquid flow. **(a)** Fluid dynamic pressure test diagrams, where (a1) represents the fluid dynamic pressure of the sample and reagents entering the liquid distribution chamber, (a2) represents the fluid dynamic pressure of the sample and reagents entering the detection chamber, and (a3) represents the fluid dynamic pressure of the sample and reagents entering the waste chamber, **(b)** Wall shear stress test diagrams, where (b1) represents the wall shear stress of the sample and reagents entering the liquid distribution chamber, (b2) represents the wall shear stress of the sample and reagents entering the detection chamber, and (b3) represents the wall shear stress of the sample and reagents entering the waste chamber, **(c)** Schematic diagram of the actual liquid flow in the chip.

Fig 4 systematically characterizes the hydrodynamic performance of each reaction chamber of the microfluidic chip, including hydrodynamic pressure (Fig 4a), wall shear stress (Fig 4b) and actual fluid flow patterns (Fig 4c). Specifically, hydrodynamic pressure (Fig 4a1) and wall shear stress (Fig 4b1) were analyzed between the sample chamber and liquid separation pool; hydrodynamic pressure (Fig 4a2) and wall shear stress (Fig 4b2) between the liquid separation pool and detection chamber; as well as hydrodynamic pressure (Fig 4a3) and wall shear stress (Fig 4b3) between the detection chamber and waste liquid pool.

Designed with a symmetrical configuration, the chip enables uniform pressure distribution and stable fluid flow within all chambers. The optimized wall shear stress contributes to the formation of a steady laminar flow field, which facilitates sufficient contact between samples and reactive substrates, improves the binding efficiency of specific antigen-antibody immune reactions, and achieves favorable actual fluid delivery performance.

Different functional layers of the chip were fabricated by 3D printing and laser cutting respectively. Compared with smooth traditional PMMA substrates, 3D-printed components possess certain microscopic surface roughness. According to the aforementioned hydrodynamic simulation and practical flow observation results, such mild surface roughness will not disrupt the stable laminar flow inside microchannels or induce turbulent flow. In addition, all inner walls of microchannels and reaction chambers were subjected to standardized blocking treatment prior to experiments, which effectively shields hydrophobic adsorption sites and markedly reduces non-specific protein adsorption caused by differences in surface morphology. Therefore, the surface roughness derived from different fabrication methods exerts no adverse effects on normal fluid transportation or the accuracy and stability of subsequent immune detection.

The above hydrodynamic analysis fully verifies the scientificity and rationality of the chip structural design, and provides solid theoretical and experimental hydrodynamic support for the stable and efficient operation of the whole multi-index simultaneous detection system.

### 3.3. Performance validation of microfluidic chips

#### 3.3.1. Evaluation of detection time parameters and accuracy for microfluidic chips.

To systematically evaluate the core performance and clinical application potential of the microfluidic chip detection technology, this study conducted a quantitative time analysis of its complete detection workflow and improved the technical evaluation system through microscopic imaging verification of control samples. It is hereby clarified that the total detection time of 24 minutes reported in this study starts after the serum sample is loaded onto the chip and does not include the time required for sample pretreatment. Temporal experimental results showed that the detection workflow of the chip comprises six key steps: sample loading (accomplished via precise pipetting, taking 2 minutes), primary antibody incubation (enabling specific immunorecognition between target analytes and specific probes, taking 7 minutes), washing treatment (effectively removing impurity components with non-specific binding, taking 2 minutes), secondary antibody incubation (enhancing detection sensitivity through a signal amplification mechanism, taking 9 minutes), secondary washing (further reducing interference caused by background fluorescence, taking 2 minutes), and signal detection and analysis (completing fluorescence signal acquisition and data preprocessing, taking 2 minutes). The total time consumption for a single run of the entire detection workflow is only 24 minutes.

Time proportion analysis indicated that the two-step immune incubation process (primary and secondary antibody incubation) accounts for 66.67% (16/24) of the total detection time, serving as the core and critical link in the entire detection workflow. In contrast, the time proportions of washing operations, sample loading, and signal detection and analysis are each 16.67% (4/24). This optimally designed “two-reaction and two-washing” detection workflow significantly improves the overall detection rate while ensuring the efficiency of specific antigen-antibody binding and the effective elimination of non-specific interference. The rapid detection cycle of only 24 minutes (calculated from the time of sample loading onto the chip) can fully meet the time requirements for clinical point-of-care testing (POCT) and high-throughput sample screening.

**Fig 5:**
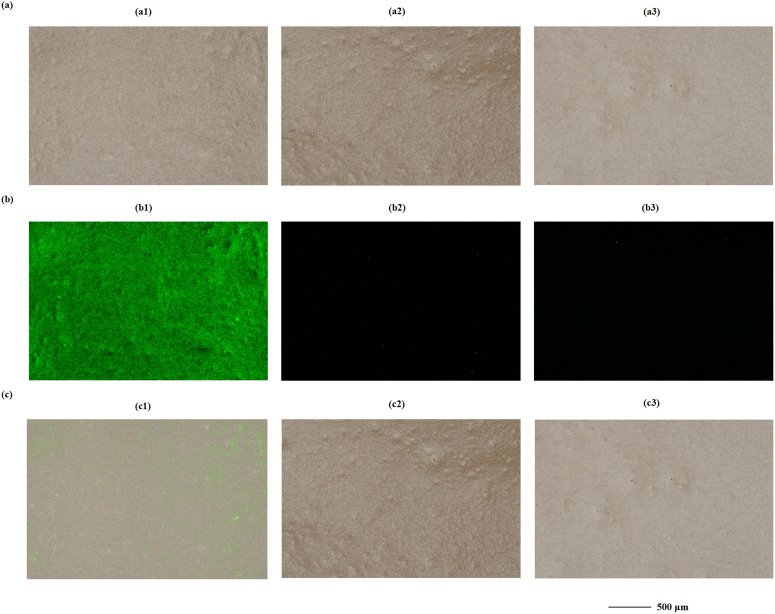
Chip performance validation results. **(a)** Bright-field microscopic images of typical positive, negative and blank samples observed under a fluorescence microscope. a1 represents the detection result of typical positive samples, a2 represents that of typical negative samples, and a3 represents that of typical blank samples. **(b)** Dark-field microscopic images of typical positive, negative and blank samples observed under a fluorescence microscope. b1 shows the result of typical positive samples, b2 shows that of typical negative samples, and b3 shows that of typical blank samples. **(c)** Merged images of bright-field and dark-field results. c1 is the merged image of positive samples, c2 is the merged image of negative samples, and c3 is the merged image of blank samples.

Microscopic imaging analysis results showed that the positive control sample exhibited strong and distinct specific fluorescence signals under dark field (Fig 5b1), while no fluorescence response was observed in the negative control (Fig 5b2) and blank control (Fig 5b3) samples. This confirms that the system possesses excellent detection specificity, which can effectively avoid false-positive results caused by non-specific interference. Bright-field observation revealed that the magnetic beads in all three groups of control samples (Fig 5a1, Fig 5a2, Fig 5a3) achieved uniform and stable enrichment and adsorption, with no aggregation or uneven binding efficiency observed. This verifies that the targeted binding and enrichment process of the target magnetic beads in the chip has good stability and controllability (see Fig 5). The bright-field and dark-field composite images of the three control groups are shown in Fig 5c1, Fig 5c2, and Fig 5c3. It can be seen that the positive control still exhibits clear fluorescence in the composite images, while weak fluorescent spots appear in the negative control and blank control. This is attributed to the fact that the chip substrate is not completely flat after 3D printing, and an extremely small amount of fluorescent microspheres are embedded in the bottom layer, which are difficult to clean thoroughly.

In summary, this study confirmed the superior performance of the microfluidic chip detection system from three core dimensions: detection timeliness, reaction specificity and operational stability, providing solid experimental evidence and technical support for its clinical translational application and the construction of a standardized detection system.

#### 3.3.2. Optimal cut-off value test.

**Fig 6:**
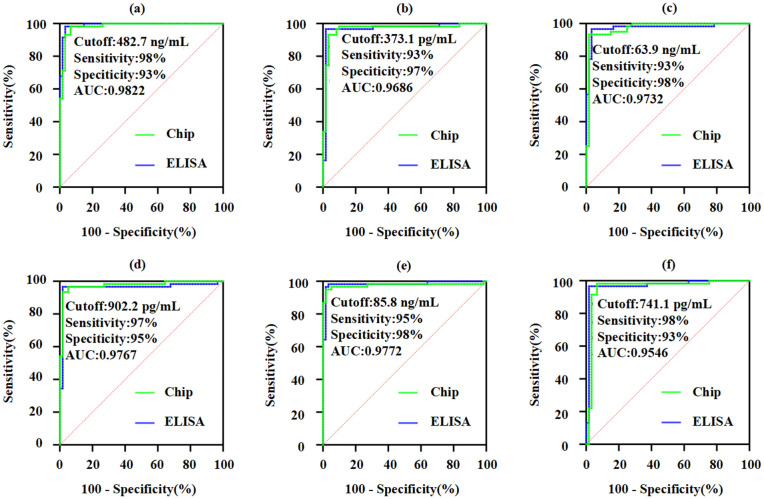
ROC analysis results of the six detection items. **(a)** ROC analysis result of MMP-9, **(b)** ROC analysis result of VEGF-A, **(c)** ROC analysis result of sN-cadherin, **(d)** ROC analysis result of OPG, **(e)** ROC analysis result of LOX; **(f)** ROC analysis result of ANG-2.

To comprehensively evaluate the detection performance of the microfluidic chip, this study performed receiver operating characteristic (ROC) curve analysis on the six core detection indicators integrated on the chip, namely matrix metalloproteinase-9 (MMP-9) (Fig 6a), vascular endothelial growth factor A (VEGF-A) (Fig 6b), soluble N-cadherin (sN-cadherin) (Fig 6c), osteoprotegerin (OPG) (Fig 6d), lysyl oxidase (LOX) (Fig 6e), and angiopoietin-2 (ANG-2) (Fig 6f). The aim was to determine the key diagnostic performance parameters of each indicator.

By calculating the detection sensitivity and specificity under different cut-off value conditions, the optimal cut-off value balancing diagnostic accuracy was screened out. Quantitative analysis was conducted on the sensitivity, specificity, and area under the curve (AUC) of each indicator. All relevant detection data have been systematically organized and summarized in Fig 6.

The ROC curve analysis of the six tumor markers fully corroborates the outstanding diagnostic performance of the microfluidic chip detection system. At the determined optimal cut-off values (MMP-9: 482.7 ng/mL, VEGF-A: 373.1 pg/mL, sN-cadherin: 63.9 ng/mL, OPG: 902.2 pg/mL, LOX: 85.8 ng/mL, ANG-2: 741.1 pg/mL), the sensitivity of each marker ranges from 93% to 98%, and the specificity remains at a high level of 93% to 98%. The area under the receiver operating characteristic curve (AUC) is between 0.9546 and 0.9822, among which MMP-9 presents the highest AUC value (0.9822), demonstrating the optimal diagnostic discrimination ability. These results indicate that the detection system features excellent capacities for positive sample identification (high sensitivity) and negative interference elimination (high specificity). The high AUC values exhibited by all six tumor markers further verify its superior diagnostic accuracy and differential value for target diseases, providing key quantitative diagnostic support for the popularization and application of this chip in practical clinical scenarios including auxiliary diagnosis and dynamic disease monitoring.

#### 3.3.3. Dose-Response Curve and Limit of Detection (LOD) Test.

**Fig 7:**
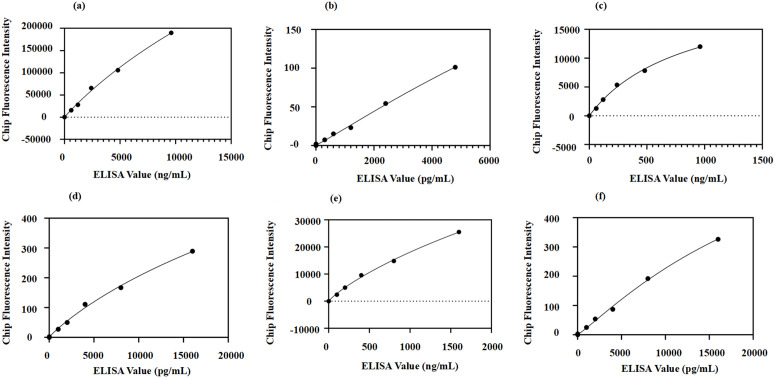
The full-concentration dose-response curve test results. **(a)** Full-concentration dose-response curve of MMP-9; **(b)** Full-concentration dose-response curve of VEGF-A; **(c)** Full-concentration dose-response curve of sN-cadherin; **(d)** Full-concentration dose-response curve of OPG; **(e)** Full-concentration dose-response curve of LOX; **(f)** Full-concentration dose-response curve of ANG-2.

In this study, the variable-slope four-parameter logistic (4PL) model was employed to fit the full concentration gradient data of six tumor markers, namely matrix metalloproteinase-9 (MMP-9, Fig 7a), vascular endothelial growth factor-A (VEGF-A, Fig 7b), soluble N-cadherin (sN-cadherin, Fig 7c), osteoprotegerin (OPG, Fig 7d), lysyl oxidase (LOX, Fig 7e), and angiopoietin-2 (ANG-2, Fig 7f), successfully establishing their respective dose-response curves. The fitting results demonstrated that the coefficient of determination (R²) for all markers ranged from 0.9976 to 0.9988, with LOX exhibiting the highest R² value (0.9988) and VEGF-A the lowest (0.9980). Regarding the parameters of the fitting equations, the bottom values (baseline) of each marker distributed between −110.0 and 1.3, the top values (plateau) ranged from 561.24 to 883765, and the hillslope values fluctuated within the range of 0.85 to 1.13. This outcome reflects the inherent differences in the immunokinetic characteristics of different tumor markers, all of which are consistent with the fitting logic of the 4PL model.

**Fig 8:**
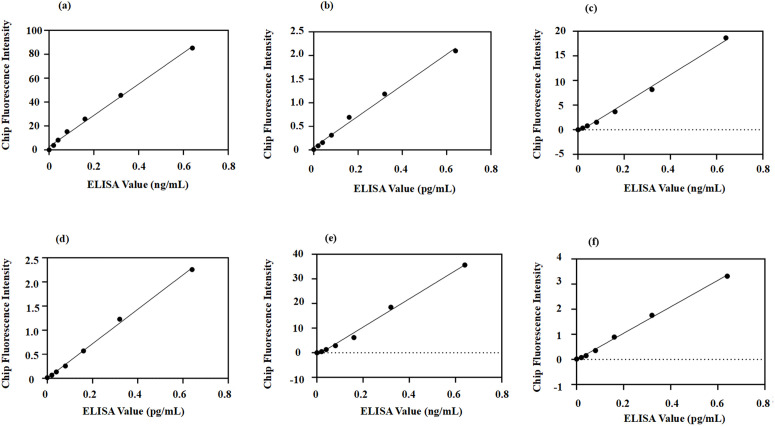
Test results of low-concentration dose-response curves. **(a)** Low-concentration dose-response curve of MMP-9; **(b)** Low-concentration dose-response curve of VEGF-A; **(c)** Low-concentration dose-response curve of sN-cadherin; **(d)** Low-concentration dose-response curve of OPG; **(e)** Low-concentration dose-response curve of LOX; **(f)** Low-concentration dose-response curve of ANG-2.

For the data within the low-concentration range (0.012 ~ 0.64 ng/mL/pg/mL), a linear model was further used for supplementary fitting. The dose-response curves of the six tumor markers are shown in Fig 8 (MMP-9 in Fig 8a, VEGF-A in Fig 8b, sN-cadherin in Fig 8c, OPG in Fig 8d, LOX in Fig 8e, and ANG-2 in Fig 8f). The results indicated that the linear fitting R² values of all markers in the low-concentration range varied from 0.9924 to 0.9979, with VEGF-A having the lowest linear R² value (0.9924) and OPG the highest (0.9977). This suggests a good linear correlation between the fluorescence signal intensity and the marker concentration within the extremely low concentration range, which can serve as a sensitive response interval for quantitative detection. The slopes of the linear equations ranged from 3.28 to 131.04, with MMP-9 showing the maximum slope (131.04), indicating its optimal signal amplification efficiency in the low-concentration range. Additionally, the absolute values of all linear equation intercepts were less than 2.73, demonstrating minimal background signal interference from the blank control and good specificity in low-concentration detection.

Through statistical analysis of blank sample signals and curve fitting calculations, the limit of detection (LOD) results of the six tumor markers revealed that VEGF-A had the lowest LOD (0.012 pg/mL) and ANG-2 the highest (0.019 pg/mL), overall demonstrating excellent low-concentration detection sensitivity. This confirms that the detection method established in this study possesses stable quantitative capability within the extremely low concentration range. Detailed data of the dose-response curves can be found in Figs 7–8, and Table 1.

**Table 1:** Statistical Results of LOD and Dose-Response Curves for the Six Detection Items.

	Item	Fitting model & Equation	*R* ^*2*^	Main applicable concentration range	Limit of detection
**Full concentration range**	MMP-9	y = -110 + 883765/1+(X/37141)^0.96^	0.9984	0.016-9600(ng/mL)	0.016(ng/mL)
	VEGF-A	y = 0.76 + 561.24/1+(X/19269)^1.09^	0.998	0.012-4800(pg/mL)	0.012(pg/mL)
	sN-cadherin	y = -12.65 + 22866.65/1+(X/875.5)^0.98^	0.9984	0.018-960(ng/mL)	0.018(ng/mL)
	OPG	y = -0.39 + 1186.61/1+(X/56135)^0.91^	0.9976	0.015-16000(pg/mL)	0.015(pg/mL)
	LOX	y = -23.67 + 147283.67/1+(X/10067)^0.85^	0.9988	0.017-1600(ng/mL)	0.017(ng/mL)
	ANG-2	y = 1.3 + 893.85/1+(X/26155)^1.13^	0.9985	0.019-16000(pg/mL)	0.019(pg/mL)
**Low-concentration range**	MMP-9	y = 131.04x + 2.7287	0.996	0.016-0.64(ng/mL)	0.016(ng/mL)
	VEGF-A	y = 3.2822x + 0.0589	0.9924	0.012-0.64(pg/mL)	0.012(pg/mL)
	sN-cadherin	y = 29.24x - 0.5149	0.995	0.018-0.64(ng/mL)	0.018(ng/mL)
	OPG	y = 3.5739x + 0.0038	0.9977	0.015-0.64(pg/mL)	0.015(pg/mL)
	LOX	y = 57.423x - 1.0322	0.9934	0.017-0.64(ng/mL)	0.017(ng/mL)
	ANG-2	y = 5.2704x - 0.0082	0.9979	0.019-0.64(pg/mL)	0.019(pg/mL)

#### 3.3.3. Repeatability test.

**Fig 9:**
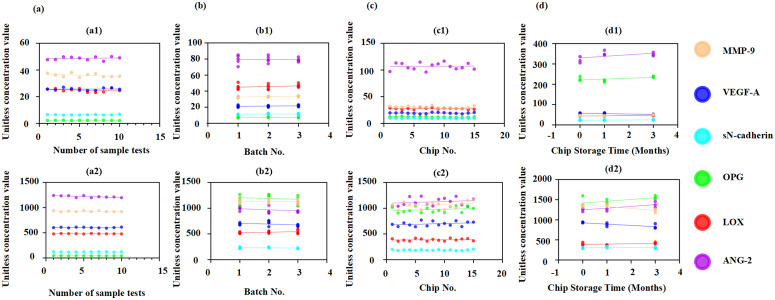
Results of chip repeatability tests. **(a)** Intra-batch repeatability results; a1 represents the intra-batch repeatability of low-concentration samples, and a2 represents that of high-concentration samples. **(b)** Inter-batch repeatability results; b1 represents the inter-batch repeatability of low-concentration samples, and b2 represents that of high-concentration samples. **(c)** Chip-to-chip repeatability results; c1 represents the chip repeatability of low-concentration samples, and c2 represents that of high-concentration samples. **(d)** Storage stability results; d1 represents the storage stability of low-concentration samples, and d2 represents that of high-concentration samples.

This study systematically evaluated the intra-batch precision (Fig 9a), inter-batch precision (Fig 9b), chip repeatability (Fig 9c), and storage stability (Fig 9d) by selecting low- and high-concentration samples of six detection indicators, including matrix metalloproteinase-9 (MMP-9), vascular endothelial growth factor A (VEGF-A), soluble N-cadherin (sN-cadherin), osteoprotegerin (OPG), lysyl oxidase (LOX), and angiopoietin-2 (ANG-2). Mean value and relative standard deviation (RSD) were adopted as the core evaluation parameters.

The results showed that the intra-batch RSD of low- and high-concentration samples for all indicators ranged from 0.88% to 5.12%: the maximum intra-batch RSD of low-concentration samples (Fig 9a1) was 5.12%, while the intra-batch RSD of high-concentration samples (Fig 9a2) did not exceed 2.21%. This indicates low dispersion of detection results within the same batch and favorable stability of the experimental reaction system.

For inter-batch precision, the RSD of each indicator for low-concentration (Fig 9b1) and high-concentration samples (Fig 9b2) varied from 3.07% to 6.48%, all within 6.50%. This demonstrated high standardization of chip preparation procedures and detection protocols, as well as excellent detection consistency across different batches.

Regarding chip repeatability, the RSD of low-concentration (Fig 9c1) and high-concentration samples (Fig 9c2) ranged from 4.87% to 6.76%, with small detection deviation among individual chips. It verified that the self-developed microfluidic chips exhibited outstanding uniformity in structural design, antibody immobilization efficiency, and immunofluorescence reaction system. Individual differences among chips exerted negligible interference on detection results, enabling parallel detection of large-scale samples.

For storage stability, the RSD of all indicators for low-concentration (Fig 9d1) and high-concentration samples (Fig 9d2) was between 4.56% and 6.16%, with minor overall fluctuation. This suggests that the chips could effectively maintain the activity of biological probes and detection performance under specified storage conditions, featuring reliable stability.

In summary, the RSD values of intra-batch precision, inter-batch precision, chip repeatability, and storage stability for the six biomarkers at both low and high concentration levels were all below 7%, which fully met the precision evaluation criteria for immunoassay methods of biomarkers. The established disc-shaped microfluidic magnetic immunofluorescence detection system possesses advantages including excellent repeatability, small batch-to-batch variation, high chip uniformity, and satisfactory storage stability. It is suitable for large-scale multiplex screening of tick-borne disease-related antibodies and biomarkers in resource-limited pastoral areas, demonstrating prominent practical application value and broad application prospects. All the overall detection results are shown in Fig 9 and Table 2.

**Table 2:** Statistical Results of Precision Assays for the Six Analytes.

Analytical items	Sample types	Intra-batch precision		Inter-batch precision		Chip repeatability		Storage stability	
		Mean value	Relative standard deviation (%)	Mean value	Relative standard deviation (%)	Mean value	Relative standard deviation (%)	Mean value	Relative standard deviation (%)
**MMP-9**	**Low value**	36.07 (ng/mL)	3.53	33.22 (ng/mL)	2.33	30.68 (ng/mL)	5.99	46.94 (ng/mL)	6.16
	**High value**	928 (ng/mL)	0.98	1132.54 (ng/mL)	3.07	1061.86 (ng/mL)	6.04	1313.67 (ng/mL)	5.89
**VEGF-A**	**Low value**	25.71 (pg/mL)	3.51	21.39(pg/mL)	4.47	19.53 (pg/mL)	5.41	55.27 (pg/mL)	5.83
	**High value**	605.1 (pg/mL)	1.3	691.76(pg/mL)	5.44	697 (pg/mL)	6.34	882.61 (pg/mL)	5.61
**sN-cadherin**	**Low value**	6.49 (ng/mL)	3.86	11.55 (ng/mL)	6.14	9.57 (ng/mL)	5.52	24.07 (ng/mL)	5.15
	**High value**	121.02 (ng/mL)	2.12	230.7 (ng/mL)	5.26	188.06 (ng/mL)	5.09	308.78 (ng/mL)	4.56
**OPG**	**Low value**	2.37 (pg/mL)	4.14	7.48 (pg/mL)	4.37	12.24 (pg/mL)	5.31	218.26(pg/mL)	5.31
	**High value**	45.17 (pg/mL)	2.21	1188.9 (pg/mL)	6.48	973.68 (pg/mL)	5.55	1475.83 (pg/mL)	4.85
**LOX**	**Low value**	24.88 (ng/mL)	5.12	44.79 (ng/mL)	6.34	27.77 (ng/mL)	4.87	45.68 (ng/mL)	5.47
	**High value**	482.05 (ng/mL)	0.88	534.51 (ng/mL)	4.83	387.77 (ng/mL)	5.76	400.17 (ng/mL)	5.87
**ANG-2**	**Low value**	48.73 (pg/mL)	2.51	79.25 (pg/mL)	5.04	106.97 (pg/mL)	6.02	339.95(pg/mL)	5.33
	High value	1218.93 (pg/mL)	1.52	970.16 (pg/mL)	5.53	1045.24 (pg/mL)	6.76	1304.2 (pg/mL)	5.06

#### 3.3.4. Specificity test.

**Fig 10:**
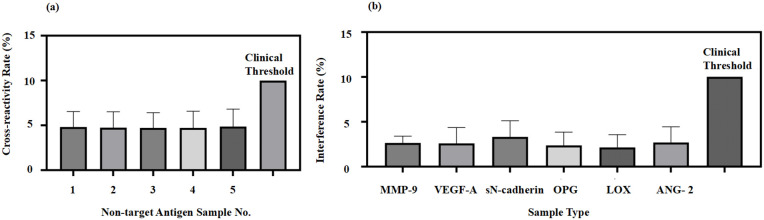
Shows the results of chip specificity tests. **(a)** Cross-reactivity test results of the chip; **(b)** Interference test results of the chip.

This study established a comprehensive verification system encompassing cross-reactivity assays, matrix interference tests, and background interference experiments to systematically evaluate the specificity of the constructed microfluidic chip detection system (integrating six detection indicators: MMP-9, VEGF-A, sN-cadherin, OPG, LOX, and ANG-2). The experimental results showed that the detection signals of all non-target antigens were significantly weaker than the specific binding signals of the corresponding target antigens, with cross-reactivity rates (CR) all below 5%—markedly lower than the clinically accepted threshold of 10%. This indicates that each detection indicator exhibits a high degree of specific binding capacity to its corresponding target molecule, with no obvious cross-reactivity observed (see Fig 10a).

In the matrix interference assessment, three types of clinically common interfering serum samples—hemolyzed samples (hemoglobin, Hb: 10–50 g/L), lipemic samples (triglyceride, TG: 5.6–22.6 mmol/L), and icteric samples (total bilirubin, TBIL: 34.2–171 μmol/L)—were mixed with target antigens for detection. The results showed that the interference rates (IR) of all indicators were controlled within 5%, meeting the clinical standard requirement for interference rate (≤ 10%). This confirms that the detection system maintains stability in complex clinical sample matrix environments without significant matrix interference (see Fig 10b).

Furthermore, the background signal values of healthy human serum samples and blank buffer samples were significantly lower than the minimum signal threshold for target antigen detection, indicating that the detection system exhibits a low level of background interference and that the non-specific binding degree of target recognition units to non-specific substances is within a controllable range (see Table 3).

**Table 3:** Specificity test results.

Sample Type	Detection Indices	Signal Value (Mean, n=5)	Positive Threshold	Below Threshold
**Healthy Human Serum (Pure Negative)**	MMP-9	39.09 (ng/mL)	482.7 (ng/mL)	Yes
	VEGF-A	16.62 (pg/mL)	373.1(pg/mL)	Yes
	sN-cadherin	4.32 (ng/mL)	63.9 (ng/mL)	Yes
	OPG	85.56 (pg/mL)	902.2(pg/mL)	Yes
	LOX	6.39 (ng/mL)	85.8 (ng/mL)	Yes
	ANG-2	96.32 (pg/mL)	741.1(pg/mL)	Yes
**Blank Buffer (PBS)**	MMP-9	28.32 (ng/mL)	482.7 (ng/mL)	Yes
	VEGF-A	9.81 (pg/mL)	373.1(pg/mL)	Yes
	sN-cadherin	4.75 (ng/mL)	63.9 (ng/mL)	Yes
	OPG	76.53 (pg/mL)	902.2(pg/mL)	Yes
	LOX	9.54 (ng/mL)	85.8 (ng/mL)	Yes
	ANG-2	111.73 (pg/mL)	741.1(pg/mL)	Yes

In conclusion, the cross-reactivity rates, matrix interference rates, and background signal levels of the microfluidic chip detection system all comply with the relevant standards for clinical applications, fully demonstrating that each target recognition unit in the system possesses excellent specific recognition performance.

#### 3.3.5. Consistency analysis results with ELISA and CLIA methods.

**Fig 11:**
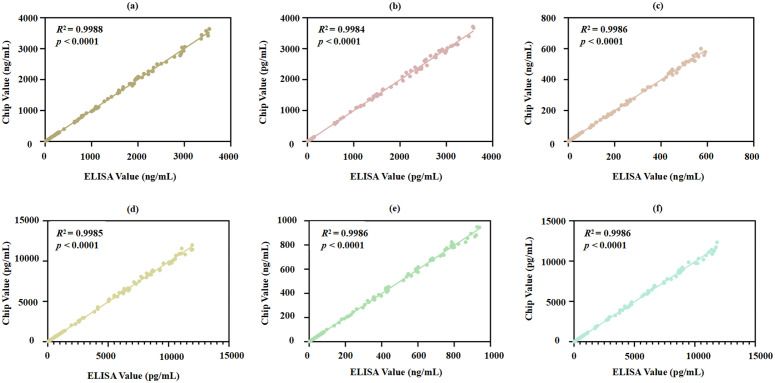
Consistency analysis results of linear scatter plots for single indicators of the chip in comparison with the ELISA method. **(a)** Linear scatter plot consistency analysis of MMP-9; **(b)** Linear scatter plot consistency analysis of VEGF-A; **(c)** Linear scatter plot consistency analysis of sN-cadherin; **(d)** Linear scatter plot consistency analysis of OPG; **(e)** Linear scatter plot consistency analysis of LOX; **(f)** Linear scatter plot consistency analysis of ANG-2.

**Fig 12:**
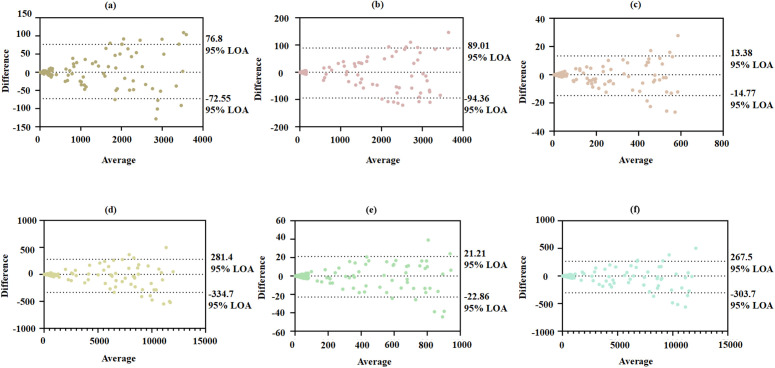
Consistency analysis results of single-index Bland-Altman plots of the chip compared with the ELISA method. **(a)** Bland-Altman consistency analysis for MMP-9; **(b)** Bland-Altman consistency analysis for VEGF-A; **(c)** Bland-Altman consistency analysis for sN-cadherin; **(d)** Bland-Altman consistency analysis for OPG; **(e)** Bland-Altman consistency analysis for LOX; **(f)** Bland-Altman consistency analysis for ANG-2.

The comprehensive results of correlation analysis, Kappa test, and Bland-Altman analysis demonstrated that the microfluidic chip detection system developed in this study exhibits excellent detection consistency and reliability compared with the ELISA detection method. Relevant detection data are detailed in Fig 11–12, and Table 4.

**Table 4:** Statistical Results of Kappa and Bland-Altman Agreement Analysis.

Item	Coincident rate		Kappa	Proportion of Samples Within 95% CI of Bland-Altman Analysis (%)
	Positive	Negative		
**MMP-9**	98.33% (59/60)	98.33% (59/60)	0.967	90% (108/120)
**VEGF-A**	100% (60/60)	98.33% (59/60)	0.983	90.8% (109/120)
**sN-cadherin**	96.67% (58/60)	100% (60/60)	0.967	94.2% (113/120)
**OPG**	98.33% (59/60)	98.33% (59/60)	0.967	92.5% (111/120)
**LOX**	98.33% (59/60)	95% (57/60)	0.933	94.2% (113/120)
**ANG-2**	100% (60/60)	95% (57/60)	0.95	93.3% (112/120)

For the foundational dataset of 720 samples, the coefficients of determination (R²) of the correlation scatter plots for the six indicators—matrix metalloproteinase-9 (MMP-9) (Fig 11a), vascular endothelial growth factor A (VEGF-A) (Fig 11b), soluble N-cadherin (sN-cadherin) (Fig 11c), osteoprotegerin (OPG) (Fig 11d), lysyl oxidase (LOX) (Fig 11e), and angiopoietin-2 (ANG-2) (Fig 11f)—between the two detection methods were all higher than 0.95, indicating an extremely strong positive linear correlation between their detection results. The Kappa values ranged from 0.8 to 1.0, further confirming the high agreement of qualitative diagnostic outcomes between the two methods. Additionally, the results of difference-based Bland-Altman analysis for the six indicators—MMP-9 (Fig 12a), VEGF-A (Fig 12b), sN-cadherin (Fig 12c), OPG (Fig 12d), LOX (Fig 12e), and ANG-2 (Fig 12f)—showed that the detection deviations of more than 90% of the samples fell within the 95% confidence interval (95% CI), and all deviations did not exceed the clinically acceptable error range.

**Fig 13:**
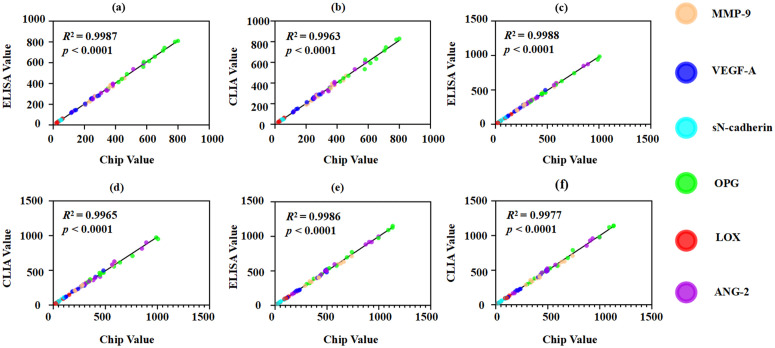
Consistency analysis of linear scatter plots for all indicators detected by the chip, compared with ELISA and CLIA methods. **(a)** Linear correlation analysis between the all-negative group and ELISA method; **(b)** Linear correlation analysis between the all-negative group and CLIA method; **(c)** Linear correlation analysis between the single-positive group and ELISA method; **(d)** Linear correlation analysis between the single-positive group and CLIA method; **(e)** Linear correlation analysis between the multi-positive group and ELISA method; **(f)** Linear correlation analysis between the multi-positive group and CLIA method.

**Fig 14:**
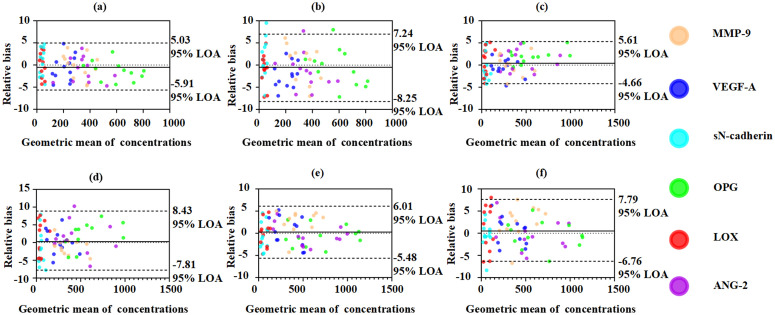
Consistency analysis results of Bland-Altman plots for all chip indicators in comparison with ELISA and CLIA methods. **(a)** Bland-Altman consistency analysis between the all-negative group and ELISA method; **(b)** Bland-Altman consistency analysis between the all-negative group and CLIA method; **(c)** Bland-Altman consistency analysis between the single-positive group and ELISA method; **(d)** Bland-Altman consistency analysis between the single-positive group and CLIA method; **(e)** Bland-Altman consistency analysis between the multi-positive group and ELISA method; **(f)** Bland-Altman consistency analysis between the multi-positive group and CLIA method.

For the 36 supplementary clinical samples, the results of consistency comparison between the microfluidic chip and two reference methods (enzyme-linked immunosorbent assay, ELISA; chemiluminescent immunoassay, CLIA) are as follows: the coefficients of determination (R²) of the correlation scatter plots for the six detection indicators were all greater than 0.99, including those of ELISA for the pure negative group (Fig 13a), CLIA for the pure negative group (Fig 13d), ELISA for the single positive group (Fig 13b), CLIA for the single positive group (Fig 13e), ELISA for the multiple positive group (Fig 13c), and CLIA for the multiple positive group (Fig 13f). This suggests an extremely high linear correlation between the detection results of different methods. The results of ratio-based Bland-Altman analysis indicated that in both reference method combinations, the proportion of sample detection deviations falling within the 95% CI for the six indicators exceeded 90%, including those of ELISA for the pure negative group (Fig 14a), CLIA for the pure negative group (Fig 14d), ELISA for the single positive group (Fig 14b), CLIA for the single positive group (Fig 14e), ELISA for the multiple positive group (Fig 14c), and CLIA for the multiple positive group (Fig 14f). The deviation levels met the clinically acceptable standards. This fully confirms that the microfluidic chip still possesses stable and reliable detection performance even in complex detection scenarios with coexisting multiple markers (see Fig 13 and Fig 14 for relevant results).

In summary, the aforementioned research results comprehensively verified the equivalence of the microfluidic chip detection system developed in this study to clinically established detection methods from three core dimensions: linear correlation, diagnostic consistency, and rationality of quantitative deviation. This provides solid methodological support for the clinical translational application of this technology and its subsequent substitution for traditional detection techniques.

### 3.4. Limitations and future perspectives

Although certain phased research outcomes have been achieved in this study, there still exist many deficiencies to be improved. Firstly, this study only completes methodological verification at a single research center, with a relatively small scale of enrolled clinical samples and limited coverage of tumor types. Multicenter, large-sample prospective clinical trials are required in follow-up studies to clarify the actual clinical diagnostic value of this detection platform in different regions, populations and various tumors.

Secondly, this study currently realizes the simultaneous combined detection of six tumor markers. Based on the molecular mechanisms of tumor occurrence and development, future research can further expand the biomarker panel, incorporate circulating tumor cells, microRNAs and other markers with high diagnostic specificity into the detection scope, and establish a more comprehensive multi-dimensional detection system. Meanwhile, novel potential biomarkers within the tumor microenvironment and substances mediating intercellular communication can be integrated into the existing detection strategy to build a more systematic and multi-level comprehensive tumor evaluation system. Markers including VEGF-A and MMP-9 selected in this study are deeply involved in tumor angiogenesis, invasion and metastasis. Combined analysis of these indicators with immunomodulatory characteristics of the tumor microenvironment, alpha-fetoprotein and its subtypes as well as other commonly used clinical serum biomarkers can comprehensively elucidate the characteristics of tumor heterogeneity. Relying on this multi-index combined detection mode, it is feasible to effectively improve the level of early tumor screening and diagnosis, enhance the accuracy of clinical prognostic judgment, and provide scientific reference for individualized diagnosis and treatment of tumor patients under complex pathological conditions [36].

Thirdly, the long-term service performance of chips such as inter-batch repeatability and shelf life, as well as large-scale mass production processes still need continuous optimization to meet the basic requirements for extensive clinical translational application. In addition, fluorescence signal collection and data analysis in this study are performed using Olympus laser confocal fluorescence microscope. Despite its excellent detection accuracy, this instrument is bulky, expensive and not portable. Therefore, the proposed detection platform is only suitable for high-precision quantitative detection in central laboratories and basic scientific research at present, and cannot be directly applied to point-of-care testing and routine primary screening in grassroots medical institutions. In subsequent research, our team will focus on developing compact, cost-effective and user-friendly portable fluorescence detection equipment to get rid of the reliance on large-scale precision research instruments. After the mature development of matched portable detection devices, this detection technology will be gradually promoted and applied in primary medical institutions and on-site rapid detection scenarios.

Finally, a comprehensive cost-benefit evaluation has not been carried out in this study. In follow-up work, it is necessary to comprehensively assess the economic benefits and popularization feasibility of this detection technology after practical clinical application combined with various clinical application scenarios.

In view of the above limitations, targeted optimization strategies will be adopted for further exploration in subsequent research. To further improve the reaction efficiency and overall detection sensitivity, structural optimization of microfluidic chip microchannels will be conducted. On the one hand, serpentine channels with staggered micro-protrusions (height: 50 μm, spacing: 100 μm) are designed to reduce mass transfer resistance via fluid chaotic mixing effect. On the other hand, the surface modification process of chip detection zones is optimized to increase the immobilization capacity and distribution uniformity of capture antibodies, so as to further improve the kinetic effect of immune reaction.

In terms of clinical research, the number of clinical verification samples will be expanded to more than 2000 cases, and cooperative experiments will be carried out in 5–8 independent research centers, including common tumors such as lung cancer, breast cancer and colorectal cancer, covering all clinical stages from stage Ⅰ to stage Ⅳ. Standardized protocols for sample collection, processing and preservation will be uniformly formulated. For example, serum samples will be stored at −80°C within 2 hours after collection to minimize detection deviations among different research centers. Moreover, the practical application efficacy of this detection system will be further verified in special populations including elderly patients over 70 years old and patients complicated with multiple underlying diseases.

In terms of chip performance optimization and industrial upgrading: firstly, highly compatible raw materials for chip preparation are selected to improve the consistency of detection results among different batches and reduce structural deformation caused by humid environment. Meanwhile, the microfabrication procedure is standardized to strictly control the channel width tolerance within ±5 μm. Secondly, a room-temperature dry protective reagent containing 5% trehalose and 0.1% bovine serum albumin is prepared, which enables the chip to maintain stable detection performance with an extremely low coefficient of variation after storage at 4°C for more than six months, thus effectively prolonging the service life of products. Thirdly, the mass production process will be continuously streamlined to reduce the unit production cost of each chip by 30% to 40%.

Furthermore, this study will explore the integrated application of artificial intelligence data analysis technology and the detection platform. Multi-biomarker detection data will be integrated to construct tumor diagnosis and prognostic evaluation models with higher accuracy, so as to provide data support for formulating individualized clinical diagnosis and treatment schemes. A complete cost-benefit investigation will also be implemented to evaluate the application value of this technology in combination with actual clinical situations. Horizontal comparisons with mainstream conventional detection techniques including enzyme-linked immunosorbent assay and chemiluminescence immunoassay will be conducted from multiple perspectives such as single-sample detection cost, labor input cost and detection turnaround time, so as to provide sufficient data support for the large-scale clinical popularization of this microfluidic combined detection technology.

## 4. Conclusions

In conclusion, this study successfully constructed a microfluidic chip-based simultaneous quantitative detection system for multi-index tumor markers. The system possesses advantages of high sensitivity, high precision, high specificity, and ultra-wide linear range in analytical performance; exhibits high consistency with traditional gold standard methods in clinical application and excellent diagnostic efficiency; and achieves rapidity, miniaturization, and low sample consumption in detection characteristics, adapting to the needs of various clinical scenarios. This technology breaks through the inherent limitations of traditional tumor marker detection technologies, providing a novel technical solution for early auxiliary diagnosis, dynamic disease monitoring, and large-scale population screening of tumors, and possesses important clinical application value and broad industrialization prospects.

## Supporting information

S1 File Raw Data Codebook. (DOCX)

S2 File Raw data. (ZIP)
