# Supplementary material for: Methodological study on simultaneous detection of 6 tumor invasion and metastasis markers including MMP-9 by microfluidic chip-based magnetic particle immunofluorescence assay
Source: PLoS One. 2026 Jun 23;21(6):e0351313. doi: 10.1371/journal.pone.0351313 (PMC13289928; doi:10.1371/journal.pone.0351313)
Supplement: S1 File — (DOCX) [file pone.0351313.s001.docx]

1. Manual Overview

This codebook focuses on the multiple verification methods adopted in the study (including ROC curve analysis, dose-response curve validation analysis, consistency analysis, and precision analysis). It clarifies the coding implications of the detection results for each method, so as to ensure the authenticity and reproducibility of the data.

1. Coding Meaning Table

2.1 Experimental Grouping Coding for dose-response curve assay

| Coding | Meaning |
| --- | --- |
| Standard concentration (pg/mL) | Standard concentration refers to the concentration of a target substance with a known concentration used to establish a standard curve, serving as the calibration benchmark for quantitative detection. |
| Fluorescence intensity | Fluorescence intensity refers to the quantitative value of the fluorescence signal captured by the detection system, reflecting the content level of the target substance (fluorescence intensity is usually positively correlated with the concentration of the target substance). |
| Fluorescence intensity of the first test | Fluorescence intensity of the first test refers to the quantitative value of the fluorescence signal obtained from the first detection of the same sample, used for verifying the consistency of repeated detections. |
| Fluorescence intensity of the second test | Fluorescence intensity of the second test refers to the quantitative value of the fluorescence signal obtained from the second repeated detection of the same sample, compared with the first test result to evaluate detection reproducibility. |
| Fluorescence intensity of the third test | Fluorescence intensity of the third test refers to the quantitative value of the fluorescence signal obtained from the third repeated detection of the same sample. The three sets of results are jointly used to calculate statistical indicators such as Mean (average value) and CV (coefficient of variation) to verify detection stability. |

| 2.2. Experimental group coding for reproducibility determination |
| --- |

| Coding | Meaning |
| --- | --- |
| Chip ID | Chip Identification (Chip ID) refers to the unique identifier assigned to each detection chip, used for tracing the individual chip in experiments, ensuring the association between detection data and the corresponding chip. |
| Detection data of low-concentration samples | Detection data of low-concentration samples refers to the original experimental data obtained from the detection of samples with low target substance concentration, including signal values and quantitative results, which are used for evaluating the detection sensitivity and reproducibility of low-concentration samples. |
| Detection data of high-concentration samples | Detection data of high-concentration samples refers to the original experimental data obtained from the detection of samples with high target substance concentration, including signal values and quantitative results, which are used for verifying the detection linearity and accuracy of high-concentration samples. |
| Chip batch ID | Chip batch Identification (Chip batch ID) is the unique identifier for distinguishing different production or experimental batches of chips. It is used for analyzing the consistency and stability of detection results among different chip batches. |
| Test run ID | Test run Identification (Test run ID) refers to the unique identifier assigned to each independent detection operation. It is applicable to repeated detection scenarios, enabling traceability of data from each individual test run and supporting reproducibility analysis. |
| Storage months | torage months refers to the duration (calculated in months) that samples or chips are stored under specified conditions before detection. It is used for evaluating the stability of samples/chips and the impact of storage time on detection results. |
|  |  |

| 2.3. Experimental group coding for consistency determination |
| --- |

| Coding | Meaning |
| --- | --- |
| Sample test result | Sample test result refers to the final quantitative or qualitative outcome obtained from the detection of a target substance in a sample. It integrates raw detection data and calibrated values, serving as the core indicator for evaluating sample status (positive/negative or concentration level). |
| Sample ID | Sample Identification (Sample ID) is a unique alphanumeric or numeric identifier assigned to each individual sample. It is used for sample traceability throughout the experiment, ensuring the association between detection results and the corresponding sample. |
| ELISA Value | ELISA Value refers to the quantitative detection result of the target substance obtained using the Enzyme-Linked Immunosorbent Assay (ELISA) method. It is typically expressed in concentration units (e.g., pg/mL, ng/mL) after calibration with a standard curve, serving as a reference for comparing with results from other detection platforms. |
| Chip Value | Chip Value refers to the quantitative detection result of the target substance obtained using a chip-based detection platform. It is a calibrated signal-derived value (corresponding to target concentration) that enables direct comparison with ELISA Value to verify the consistency and correlation between different detection methods. |

| 2.4. Experimental group coding for specificity determination |
| --- |

| Coding | Meaning |
| --- | --- |
| Serial number of non-target antigen solution detection results | A unique identifier assigned to the test outcomes of non-target antigen solutions, used for tracing, filing and differentiating individual detection records in the coding book. |
| Hemoglobin concentration of hemolyzed specimens (g/L) | The quantitative value of hemoglobin content in hemolyzed samples, which is a key test indicator recorded in the coding book for evaluating specimen quality or related physiological/pathological conditions. |
| Test batch result of specimens | The integrated test outcome of a group of specimens processed in the same test batch, including overall pass/fail status, batch-specific data statistics or comprehensive evaluation, which is recorded in the coding book for batch-wise traceability and quality control. |
| Total bilirubin concentration in specimens (μmol/L) | The measured concentration of total bilirubin in the tested specimens, a critical biochemical indicator documented in the coding book for clinical diagnosis or specimen assessment. |
| Serial number of test runs | A sequential identification number assigned to each independent test run (including equipment operation cycle, batch testing process or single detection procedure), used in the coding book to track the order and details of each test execution. |

| 2.5. Experimental group coding for ROC curve analysis |
| --- |

| Coding | Meaning |
| --- | --- |
| Sample ID | Unique alphanumeric code assigned to each individual sample for tracking and differentiation in experimental processes. |
| ELISA Value | Numerical value reflecting the concentration or reactivity of the target analyte (e.g., antigen, antibody) measured by the ELISA method, typically expressed in appropriate units. |
| Chip fluorescence intensity | Quantitative measurement of the fluorescence signal emitted by probes on the microarray chip after hybridization, indicating the abundance or binding affinity of the target biomolecule in the sample. |
